# Lysosomotropic drugs activate TFEB via lysosomal membrane fluidization and consequent inhibition of mTORC1 activity

**DOI:** 10.1038/s41419-018-1227-0

**Published:** 2018-12-13

**Authors:** Benny Zhitomirsky, Anna Yunaev, Roman Kreiserman, Ariel Kaplan, Michal Stark, Yehuda G. Assaraf

**Affiliations:** 1 The Fred Wyszkowski Cancer Research Laboratory, Technion-Israel Institute of Technology, Haifa, Israel; 2 Department of Biology, Technion-Israel Institute of Technology, Haifa, Israel

## Abstract

Transcription factor EB (TFEB) is a master transcriptional regulator playing a key role in lysosomal biogenesis, autophagy and lysosomal exocytosis. TFEB activity is inhibited following its phosphorylation by mammalian target of rapamycin complex 1 (mTORC1) on the surface of the lysosome. Phosphorylated TFEB is bound by 14-3-3 proteins, resulting in its cytoplasmic retention in an inactive state. It was suggested that the calcium-dependent phosphatase calcineurin is responsible for dephosphorylation and subsequent activation of TFEB under conditions of lysosomal stress. We have recently demonstrated that TFEB is activated following exposure of cancer cells to lysosomotropic anticancer drugs, resulting in lysosome-mediated cancer drug resistance via increased lysosomal biogenesis, lysosomal drug sequestration, and drug extrusion through lysosomal exocytosis. Herein, we studied the molecular mechanism underlying lysosomotropic-drug-induced activation of TFEB. We demonstrate that accumulation of lysosomotropic drugs results in membrane fluidization of lysosome-like liposomes, which is strictly dependent on the acidity of the liposomal lumen. Lysosomal accumulation of lysosomotropic drugs and the consequent fluidization of the lysosomal membrane, facilitated the dissociation of mTOR from the lysosomal membrane and inhibited the kinase activity of mTORC1, which is necessary and sufficient for the rapid translocation of TFEB to the nucleus. We further show that while lysosomotropic drug sequestration induces Ca^2+^ release into the cytoplasm, facilitating calcineurin activation, chelation of cytosolic Ca^2+^, or direct inhibition of calcineurin activity, do not interfere with drug-induced nuclear translocation of TFEB. We thus suggest that lysosomotropic drug-induced activation of TFEB is mediated by mTORC1 inhibition due to lysosomal membrane fluidization and not by calcineurin activation. We further postulate that apart from calcineurin, other constitutively active phosphatase(s) partake in TFEB dephosphorylation and consequent activation. Moreover, a rapid export of TFEB from the nucleus to the cytosol occurs upon relief of mTORC1 inhibition, suggesting that dephosphorylated TFEB constantly travels between the nucleus and the cytosol, acting as a rapidly responding sensor of mTORC1 activity.

## Introduction

Small molecules with hydrophobic weak base properties markedly accumulate in lysosomes via a mechanism known as ion trapping; due to their hydrophobic nature, these compounds cross the plasma membrane and lysosomal membrane via diffusion. However, upon encountering the acidic lumen of the lysosome, the weakly basic residues of these compounds become protonated, preventing them from crossing the lysosomal membrane back to the cytoplasm^1–4^. We have recently demonstrated that lysosomal sequestration of anticancer drugs contributes to cancer drug resistance by reducing the concentration of these drugs at their cellular target sites and by activating lysosomal exocytosis, resulting in the extrusion of the sequestered drugs from the cells^2,5–7^. Furthermore, lysosomal sequestration of anticancer drugs triggered lysosomal biogenesis via activation and nuclear translocation of transcription factor EB (TFEB)^5^, the master regulator of lysosomal biogenesis, and an activator of autophagy and lysosomal exocytosis^8–11^. We have shown that drug naïve cells with intrinsically higher lysosome number per cell, sequester lysosomotropic chemotherapeutics more efficiently away from their target sites when compared to cells with low-lysosome number per cell, which contributes to an enhanced intrinsic resistance to lysosomotropic chemotherapeutics. Based on these collective findings, we have proposed a novel model for drug-induced lysosome-mediated acquired drug resistance, in which lysosomal accumulation of anticancer drugs induces TFEB-mediated lysosomal biogenesis, an elevation in lysosome number per cell, and consequent chemoresistance due to increased lysosomal drug sequestration^5^.

TFEB was shown to control lysosomal biogenesis via the transcriptional activation of genes from the coordinated lysosomal expression and regulation (CLEAR) pathway, upon its translocation into the nucleus^10,12^. The activity of TFEB is inhibited via its phosphorylation on Ser^211^ by mammalian target of rapamycin complex 1 (mTORC1)^13^. mTORC1, a serine/threonine protein kinase which responds to cellular stress cues such as deprivation of amino acids, low oxygen tension, changes in energy availability and various growth factors, is responsible for the promotion of cell growth by regulating anabolic and catabolic processes. mTORC1 is one of two complexes in which mTOR is a core component, the other being mTORC2, which regulates metabolism and cell survival and was shown to effect the actin cytoskeleton, but has no effect on TFEB phosphorylation and inactivation^14,15^. The phosphorylation of TFEB by mTORC1 enables its binding by 14-3-3 proteins to TFEB, resulting in its retention in the cytoplasm. mTORC1 was shown to phosphorylate TFEB on the lysosomal surface, while TFEB was shown to be recruited to the lysosomal surface via interaction with Rag guanosine triphosphatases (GTPases)^16,17^. TFEB dephosphorylation and consequent activation is attributed to the calmodulin-regulated calcium-dependent protein phosphatase calcineurin. It was suggested that calcineurin dephosphorylates TFEB upon Ca^2+^ release from the lysosome through the Ca^2+^ channel mucolipin 1 (MCOLN1)^9^. It was postulated that cues inducing lysosomal stress, including starvation and physical exercise, activate MCOLN1, resulting in an efflux of Ca^2+^ from the lysosome, a cytoplasmic increase in Ca^2+^ concentrations and consequent activation of calcineurin. The latter dephosphorylates TFEB, leading to TFEB translocation to the nucleus, and consequent activation of lysosomal biogenesis and autophagy^9^.

The lysosomotropic drug (LD) siramesine exerts lysosomal membrane permeabilization (LMP) and lysosomal leakage, and provokes reactive oxygen species generation via a detergent-like activity^18,19^. However, the impact of LD sequestration on the lysosomal membrane remains unclear. Herein, we explored this impact and the molecular mechanism underlying drug-induced activation of TFEB. We present the first evidence suggesting that LDs inhibit the kinase activity of mTORC1 via fluidization of the lysosomal membrane, and that this inhibitory effect is the driving force behind the drug-induced activation of TFEB that is independent of calcineurin, resulting in elevated lysosomal biogenesis, lysosomal exocytosis, and autophagy.

## Results

### LDs induce lumen acidity-dependent membrane fluidization of lysosome-like liposomes and inhibit mTORC1 kinase activity

**Fig. 1:**
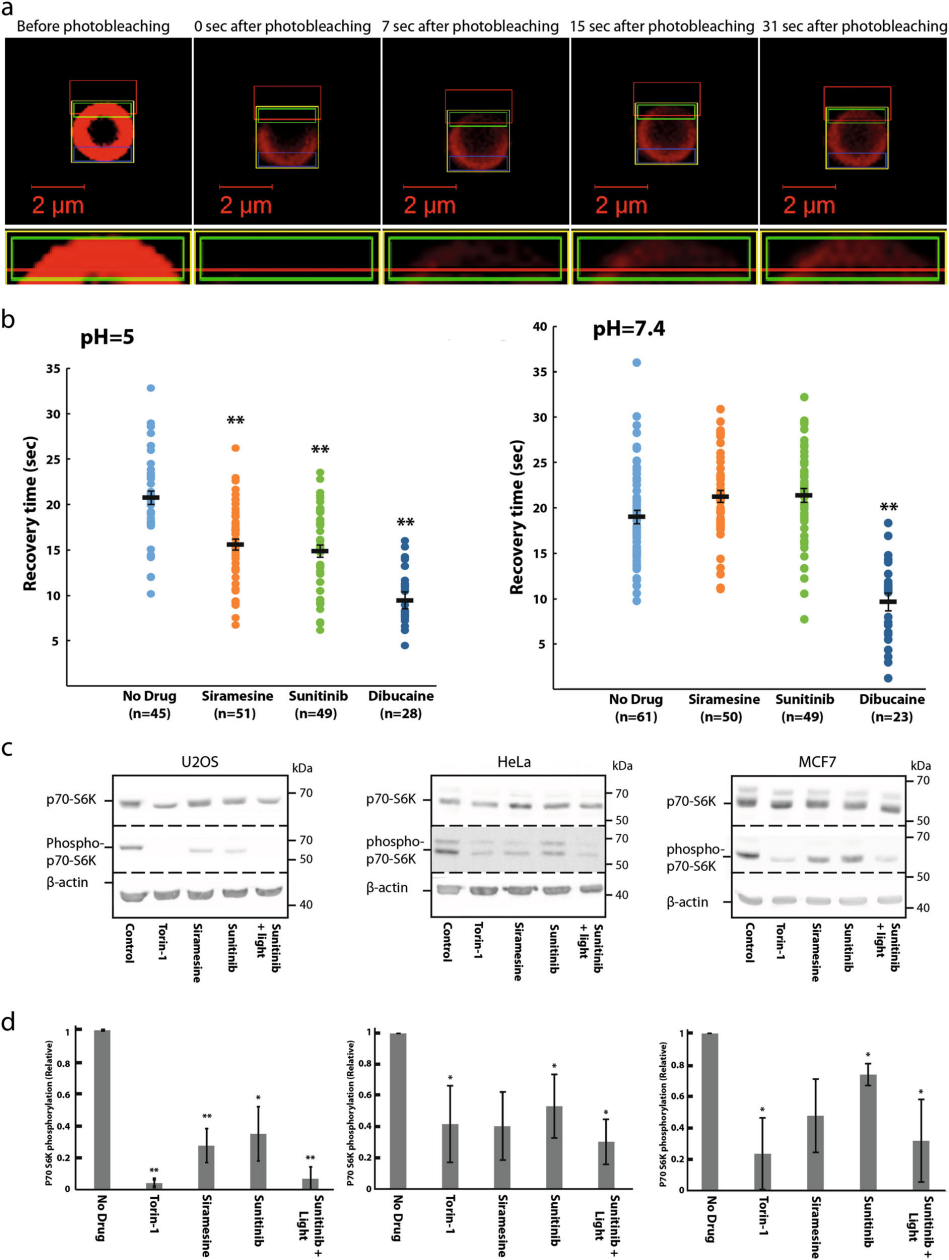
Siramesine and sunitinib induce lumen acidity-dependent membrane fluidization in liposomes and inhibit mTORC-1-mediated phosphorylation of p70-S6 kinase. **a**, **b** For FRAP analysis, red fluorescent liposomes were loaded with an acidic buffer (pH 5.0) or a neutral buffer (pH 7.4), and incubated for 30 min with siramesine (10 µM), sunitinib (10 µM), dibucaine (2 mM), or without any drug. A segment of the liposome was photobleached by a Zeiss LSM 700 confocal microscope. The fluorescence intensity of the photobleached area and an unbleached area, used as reference, were followed for 2 min by the confocal microscope. Fluorescence recovery time was calculated using integrated settings in the confocal microscope software as described in Materials and methods. **p Value < 0.01—two-tailed student's *t* test. Error bars indicate standard error of the mean. Representative early time-course points of fluorescence recovery snapshots are shown to exemplify the rapid red fluorescence recovery in the photobleached zone. **c** U2OS, MCF7, and HeLa cells were treated for 4 hr with Torin-1 (10 nM), siramesine (10 µM), sunitinib (10 µM), as well as sunitinib (10 µM) with 1 hr of illumination. Cytosolic proteins were extracted from the cells, and WB analysis was performed using, p70-S6K and phospho-p70-S6K antibodies. A β-actin antibody was used to assess actual protein loading. **d** Band intensity was quantified using ImageJ software. Phosphorylation levels were determined by dividing the level of phospho-p70-S6K by the levels of total p70-S6K for each treatment. *p value < 0.05, **p value < 0.01 using two-tailed Student's *t* test

We employed the fluorescence recovery after photobleaching (FRAP) assay to explore the impact of LDs on membrane fluidity of lysosomal-like liposomes. Liposomes that mimic the lipid composition of the lysosomal membrane were prepared^20^ (see Materials and methods). Lissamine rhodamine-tagged lipids were integrated into the liposomes, hence marking their membrane with red fluorescence. Liposomes were loaded with either an acidic (pH 5.0) or a neutral (pH 7.4) buffer solution. Liposomes were preincubated for 30 min with the LDs: sigma-2 receptor ligand siramesine (10 μM) and the receptor tyrosine kinase (RTK) inhibitor sunitinib (10 μM), currently used for the treatment of renal cell cancer. The local anesthetic membrane fluidizing agent dibucaine (2 mM) was used as a positive control. Following preincubation with the drugs, a segment of the liposomal membrane was photobleached, and the fluorescence recovery time via diffusion of rhodamine-tagged lipids from the unbleached segment into the bleached zone was determined (Fig. 1a); a rapid fluorescence recovery was observed in the photobleached zone. A significant ~25% reduction in the fluorescence recovery time was observed after incubation with siramesine and sunitinib in liposomes loaded with an acidic buffer solution, indicating liposome membrane fluidization. Remarkably, no decrease in the fluorescence recovery time was recorded after incubation with siramesine and sunitinib in liposomes loaded with a neutral buffer solution. Expectedly, dibucaine markedly decreased the fluorescence recovery time independently of liposomal pH (Fig. 1b). These results demonstrate for the first time that hydrophobic weakly basic LDs induce a lumen pH-dependent membrane fluidization of acidic organelles. It is important to note that the lysosomal membrane is rather complex as it contains multiple integral proteins and various lipids when compared to the liposomes used in this FRAP analysis. As these differences might possibly have an effect on the extent of drug-induced membrane fluidization, additional dedicated studies are warranted to further determine the extent and significance of drug-induced membrane fluidization on lysosomes.

Since the lysosomal membrane was shown to act as a hub for signal transduction from the lysosome into the nucleus^10^, we postulated that this drug-induced membrane fluidization might play a significant role in the previously reported drug-induced lysosomal signaling^5,6^. We specifically postulated that lysosomal accumulation of LDs and consequent membrane fluidization, might disrupt the kinase activity of mTORC1, which resides on the lysosomal membrane. We hence performed Western Blot (WB) analysis to determine the phosphorylation level of the established mTORC1 substrate p70S6 kinase (p70-S6K), following exposure of human osteosarcoma U2OS cells, breast cancer MCF-7 cells as well as cervical cancer HeLa cells to siramesine and sunitinib. These LDs, as well as Torin-1, a potent mTOR inhibitor, blocked mTORC1 kinase activity in all three human tumor cell lines as evident from the significantly reduced levels of p70-S6K phosphorylation (Fig. 1c). When cells loaded with sunitinib were illuminated to induce a lysosome-mediated photodynamic effect as previously reported^21^, inhibition of mTORC1 kinase activity was further enhanced. This result, which represents the first indication of LD-induced inhibition of mTORC1 activity, suggests that drug-induced activation of TFEB is presumably a result of inhibition of mTORC1-mediated TFEB phosphorylation.

### LDs induce the release of mTOR from the surface of lysosomes

**Fig. 2:**
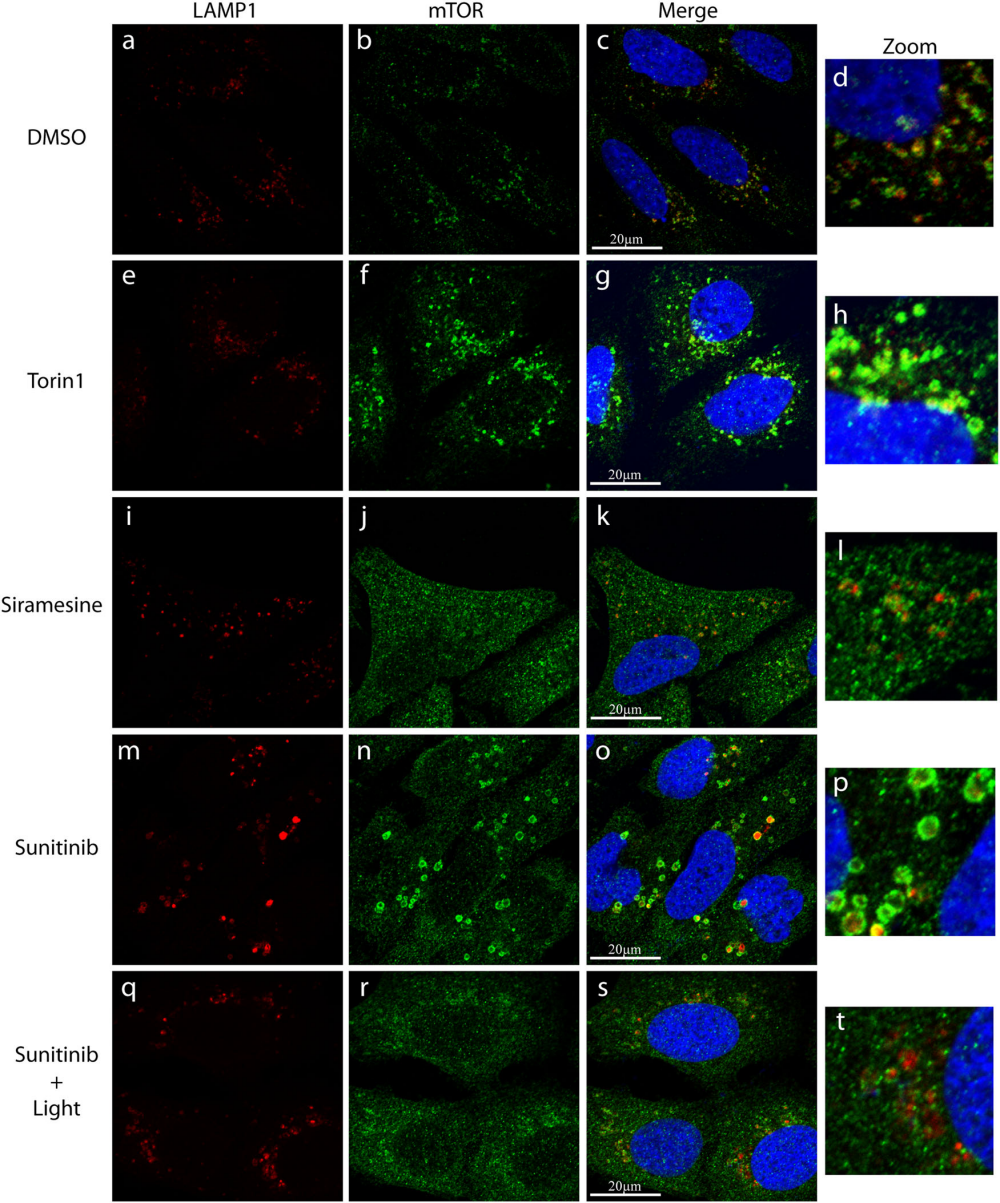
Immunofluorescence microscopy exploring the association of mTOR to lysosomes, following drug exposure. U2OS cells were incubated with the indicated drugs for 3 hr and then subjected to immunofluorescence microscopy as detailed under Materials and methods. The localization of mTOR (green fluorescence) was followed together with LAMP1 (red fluorescence) which was used as a lysosomal marker. The right row depicts blow-ups of areas from the merged photos showing the (dis)association of mTOR and LAMP1. Nuclei were stained with the blue DNA dye Hoechst 33342. Cells were visualized by scanning confocal microscopy at a ×63 magnification. All fields are representative of at least three independent experiments

It was previously demonstrated that the recruitment of mTORC1 to the lysosomal membrane is essential for its activation^22,23^. We thus postulated that the membrane fluidization identified in our current study, might lead to the dissociation of mTORC1 from the lysosomal membrane, resulting in inhibition of mTORC1 kinase activity. To explore this hypothesis, U2OS cells were treated with either the vehicle (0.1% DMSO), Torin-1 (10 nM), siramesine (10 µM), sunitinib (10 µM), or sunitinib with illumination for 3 hr. An immunofluorescence assay was used to follow mTOR and the lysosomal membrane protein LAMP1. In control cells incubated in the absence of drugs, mTOR was highly localized on the lysosomal membrane, as evident from its co-localization with LAMP1 and its relatively low levels in the cytosol (Fig. 2a–d). The mTOR inhibitor Torin-1, which directly and specifically inhibits mTORC1 activity via an ATP-comparative mechanism^24^, did not modulate the lysosomal localization of mTOR (Fig. 2e–h). It is noteworthy, however, that Torin-1 treatment resulted in an apparent increase in the total mTOR levels within the cells. We postulate that this might be indicative of activation of a compensatory translational mechanism due to reduced mTOR activity.

Remarkably, siramesine treatment resulted in a marked release of mTORC1 from the lysosomal membrane as evident from the reduced co-localization between mTOR and LAMP1 along with a significant increase in cytosolic mTOR levels (Fig. 2i–l). Sunitinib treatment also resulted in a significant increase in cytosolic mTOR levels, indicative of mTOR release from the lysosomal membrane. However, the sunitinib-induced effect on mTOR localization seemed to be to a lesser extent when compared to that of siramesine, as significant levels of mTOR were still co-localized with LAMP-1 on the lysosomal membrane in sunitinib-treated cells (Fig. 2m–p). Intriguingly, illumination of sunitinib-treated cells resulted in the dissociation of the remaining mTOR from the lysosomal membrane (Fig. 2q–t). As siramesine was previously shown to induce LMP^18^ and illuminating sunitinib-loaded cells results in photodestruction of lysosomes^21^, we postulate that the release of mTORC1 from the lysosomal membrane was a result of the combined effect of lysosomal membrane fluidization and LMP.

### LDs induce a rapid translocation of TFEB into the nucleus and transcriptional activation of CLEAR pathway genes

**Fig. 3:**
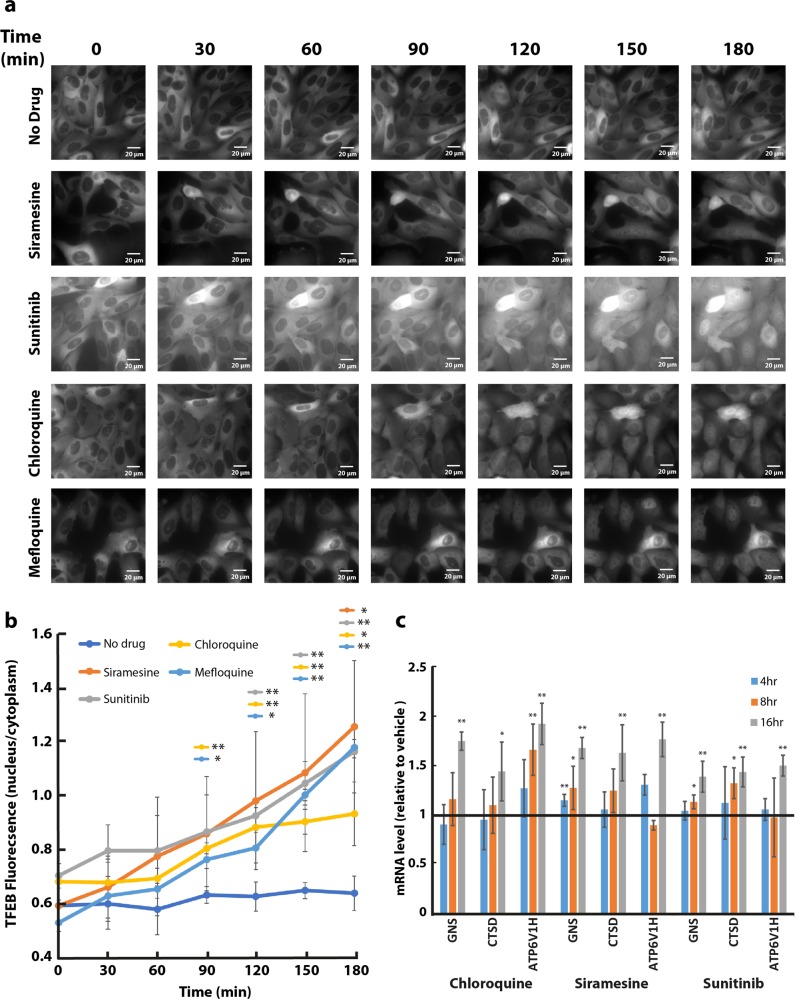
Lysosomotropic drugs induce a rapid translocation of TFEB into the nucleus and activation of the CLEAR network. **a** U2OS-TFEB-eGFP cells were incubated with siramesine (10 µM), sunitinib (10 µM), chloroquine (100 µM), or mefloquine (10 µM) for 3 hr. TFEB-eGFP was visualized by an InCell Analyzer fluorescence microscope every 30 min. **b** The ratio between nuclear and cytoplasmic TFEB-eGFP levels was determined by staining nuclei with Hoechst 33342 prior to drug exposure (2 µg/ml, staining not shown), and image analysis by InCell investigator software. **c** U2OS cells were incubated with siramesine (10 µM), sunitinib (10 µM), and chloroquine (100 µM) for 4, 8, or 16 hr, followed by RNA purification. Real-time PCR analysis was performed to determine the levels of GNS, CTSD, and ATP6V1H mRNA. *p value < 0.05, **p value < 0.01—two-tailed Student's *t* test. Error bars indicate standard deviation

To follow the subcellular localization of TFEB, U2OS, and MCF-7 cells were stably transfected with TFEB-eGFP (U2OS-TFEB-eGFP and MCF-7-TFEB-eGFP, respectively). To determine the impact of LD-dependent lysosomal membrane fluidization on the subcellular localization of TFEB, U2OS-TFEB-eGFP, and MCF-7-TFEB-eGFP cells were exposed to various LDs including siramesine, sunitinib, and the anti-malarial drugs chloroquine and mefloquine. Time-lapse fluorescence microscopy revealed that these drugs induced a rapid nuclear translocation of TFEB in both tumor cell lines, which was evident as early as 90 min after drug exposure, and further increased during the following hours of incubation (Fig. 3a, b and Supplementary Fig. S1). The impact of this nuclear translocation of TFEB on the transcription of CLEAR genes was determined by quantitative real-time polymerase chain reaction (PCR) analysis for glucosamine (N-acetyl)-6-sulfatase (GNS), cathepsin-D (CTSD), and V-type proton ATPase subunit HATPV1H (ATP6V1H) (Fig. 3c). An increase in the expression levels of these genes (up to twofold) was observed after exposure of U2OS cells to siramesine (10 μM), sunitinib (10 μM), and chloroquine (100 μM), indicating that drug-induced nuclear translocation of TFEB results in transcriptional activation of the CLEAR network (Fig. 3c). WB analysis was used to confirm that, in agreement with previous studies^15^, following drug treatment, TFEB was translocated to the nucleus in its unphosphorylated form (Supplementary Fig. S2). Following exposure to siramesine, TFEB levels were markedly increased in the nucleus and its molecular weight was slightly decreased presumably as a result of its dephosphorylation (Fig. S2, compare lane 4 to lanes 2 and 3, respectively). This was accompanied by a decrease in the cytosolic levels of TFEB. In concordance with the results of our time-lapse fluorescence microscopy analyses, sunitinib displayed a less pronounced effect on TFEB translocation to the nucleus, however, illuminating the cells intensified this nuclear translocation (Fig. S2, compare lanes 6 and 8 to lane 2).

### LD-induced activation of TFEB is independent of calcineurin activity

Activation of TFEB was suggested to be induced via Ca^2+^ release from the lysosome, and consequent activation of the Ca^2+^-dependent serine/threonine phosphatase calcineurin^9^. To determine whether LDs induce Ca^2+^ release into the cytoplasm, U2OS cells were preincubated with the cell-permeable fluorescent calcium-binding dye Fluo-8-AM, followed by treatment with siramesine or chloroquine for 15 min, during which Fluo-8-AM fluorescence was visualized by time-lapse fluorescence microscopy. Drug treatment induced pulses of Ca^2+^ release into the cytoplasm which was evident by rapid transient pulse increases in Fluo-8-AM fluorescence (Supplementary Videos 1a–c). These results suggest that treatment with siramesine and chloroquine, provokes the release of Ca^2+^ from the Ca^2+^-rich lysosomal lumen into the cytoplasm. Pretreatment with the cell permeable Ca^2+^ chelator BAPTA-AM was successfully used to chelate the Ca^2+^ released into the cytoplasm, following treatment with siramesine and chloroquine (Supplementary Videos 1d, e).

**Fig. 4:**
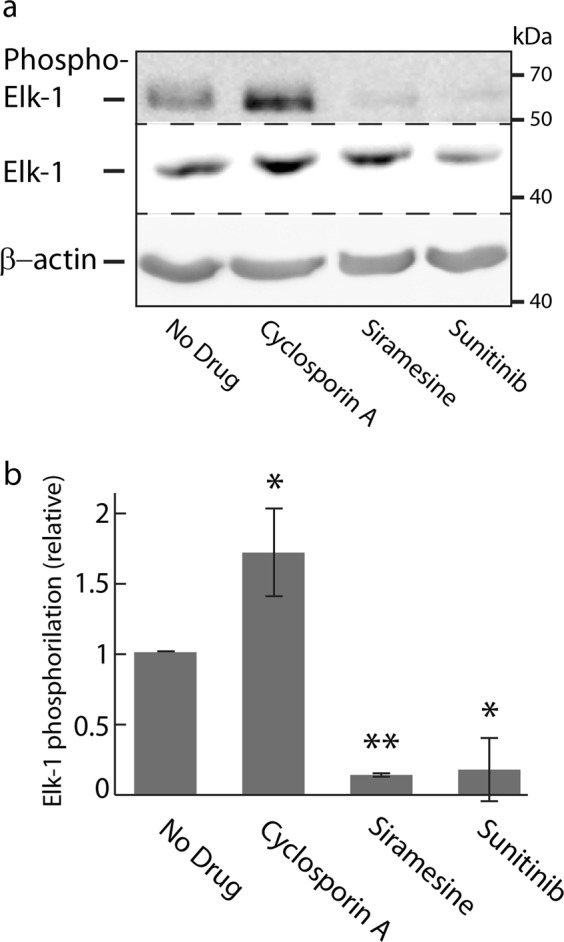
Lysosomotropic drugs activate calcineurin, resulting in dephosphorylation of its substrate Elk-1. **a** U2OS cells were treated with cyclosporin A (10 µM), siramesine (10 µM), or sunitinib (10 µM) for 4 hr. Nuclear proteins were extracted from the cells, and WB analysis was performed using Elk-1 and phospho-Elk-1 (Ser383)-specific antibodies. A β-actin-specific antibody was used to verify equal loading. **b** Band intensity was quantified using ImageJ software. Phosphorylation levels were determined by dividing the level of phospho-Elk-1 by the levels of total Elk-1 for each treatment. *p value < 0.05, **p value < 0.01—two-tailed Student's *t* test

**Fig. 5:**
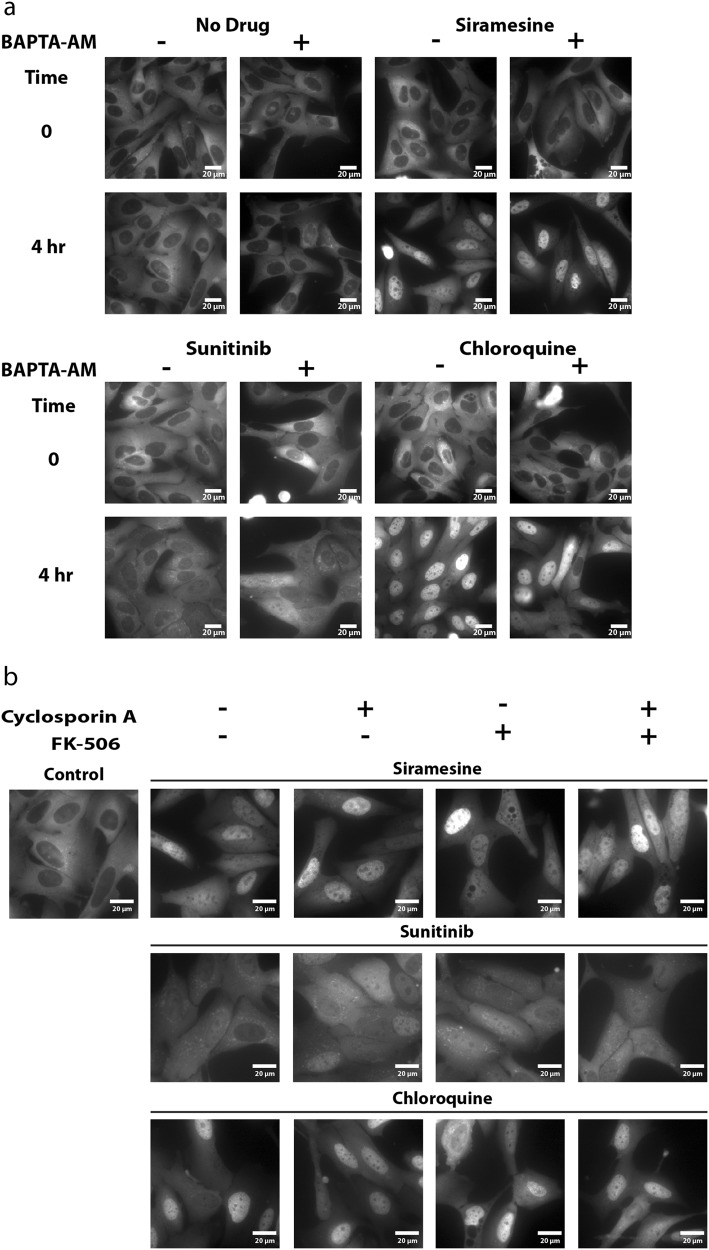
Inhibition of calcineurin does not prevent drug-induced nuclear translocation of TFEB. **a** U2OS-TFEB-eGFP cells were preloaded with BAPTA-AM (10 µM, 30 min), washed and incubated with siramesine (10 µM), sunitinib (10 µM), or chloroquine (100 µM) for an additional 4 hr. **b** U2OS-TFEB-eGFP cells were co-treated with or without the calcineurin inhibitors CsA (10 µM), FK-506 (5 µM), or both, as well as with siramesine (10 µM), sunitinib (10 µM), or chloroquine (100 µM). TFEB-eGFP was visualized using an InCell Analyzer fluorescence microscope

The phosphorylation state of the transcription factor Elk-1, a bona fide substrate of calcineurin^25^, was examined by WB analysis, to determine whether said release of Ca^2+^ into the cytoplasm leads to calcineurin activation. Consistent with the previous result, exposure of U2OS cells to siramesine and sunitinib resulted in increased dephosphorylation of Elk-1, suggesting that the above reported increase in cytosolic Ca^2+^ levels resulted in the activation of calcineurin phosphatase activity (Fig. 4). Expectedly, the calcineurin inhibitor cyclosporin A (CsA) elevated Elk-1 phosphorylation levels, due to inhibition of calcineurin phosphatase activity (Fig. 4). We thus postulated that pretreating U2OS-TFEB cells with BAPTA-AM will prevent drug-induced nuclear translocation of TFEB, due to chelation of Ca^2+^, and consequent retention of calcineurin in an inactive state. Surprisingly, pretreatment of U2OS-TFEB-eGFP cells with BAPTA-AM did not prevent nuclear translocation of TFEB following exposure to siramesine, sunitinib, or chloroquine (Fig. 5a). To further determine whether or not calcineurin activity is required for the drug-induced activation of TFEB, U2OS-TFEB-eGFP cells were co-treated with the LDs siramesine, sunitinib and chloroquine, along with the established calcineurin inhibitors CsA and FK-506 (tacrolimus). In agreement with the previous result, direct pharmacologic inhibition of calcineurin did not prevent LD-induced translocation of TFEB into the nucleus (Fig. 5b). These results suggest that calcineurin activity is not required for the dephosphorylation and activation of TFEB, and that a different, Ca^2+^-independent phosphatase(s) may possibly dephosphorylate TFEB and facilitate its nuclear translocation under these conditions.

### LD-induced activation of TFEB is caused by mTORC1 inhibition

To determine whether or not inhibition of mTORC1 is sufficient to induce nuclear TFEB translocation, U2OS-TFEB-eGFP cells were exposed to increasing concentrations of Torin-1, an established mTOR inhibitor^24^. Nanomolar concentrations of Torin-1 were sufficient to induce a marked nuclear TFEB translocation as early as 12 min after drug exposure (Supplementary Fig. S3). We therefore postulated that this rapid nuclear TFEB translocation following inhibition of mTORC1 suggests a constitutive dephosphorylation of TFEB, which, under normal conditions, is balanced by the constitutive phosphorylation of TFEB by mTORC1. Disrupting this equilibrium by inhibition of mTORC1, results in a rapid shift towards an unphosphorylated TFEB state, resulting in its rapid nuclear translocation.

**Fig. 6:**
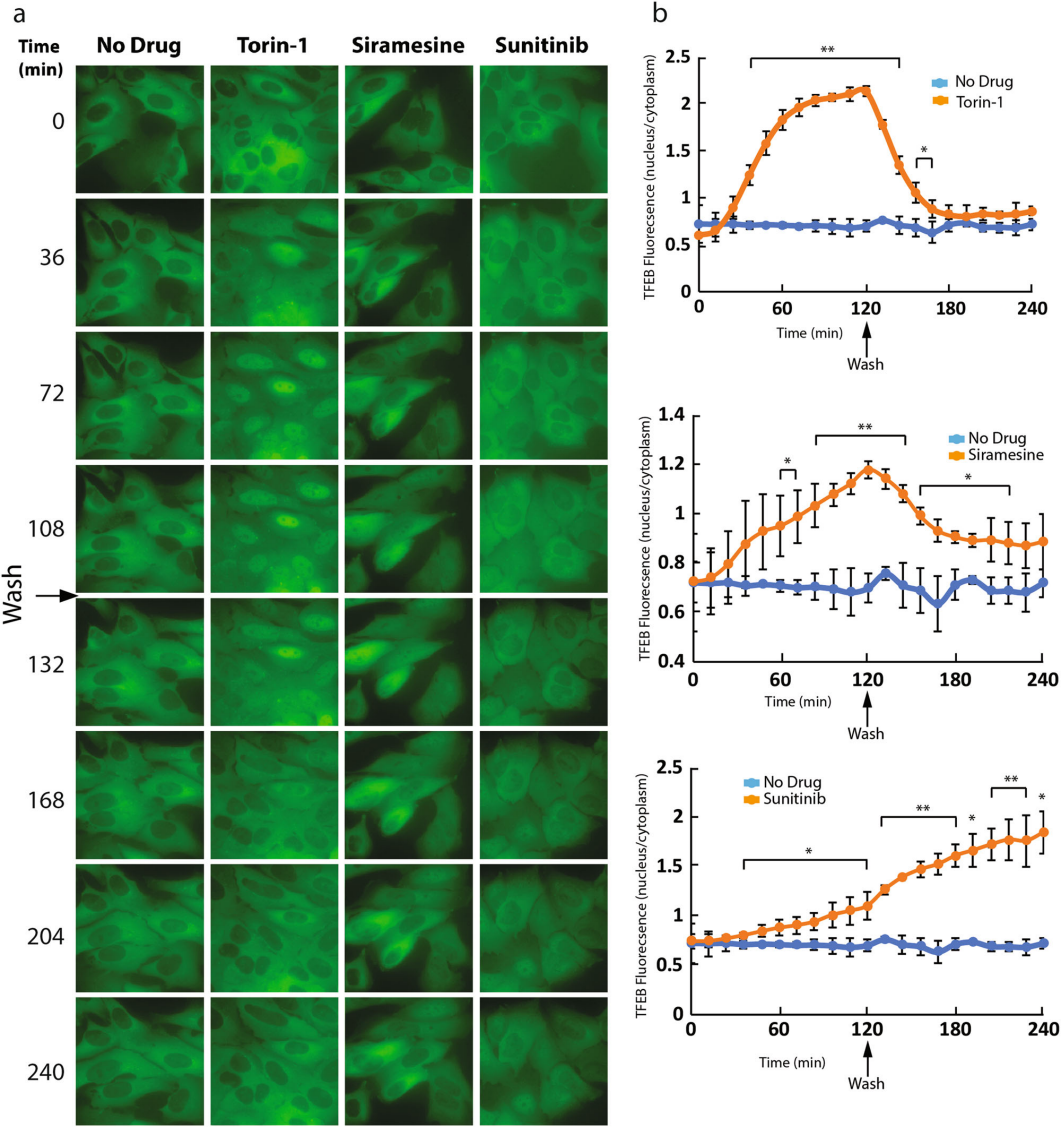
Cytoplasmic localization of TFEB is restored after removal of Torin-1 and siramesine, but not after removal of sunitinib from the growth medium. **a** U2OS-TFEB-eGFP cells were treated with Torin-1 (10 nM), siramesine (10 µM), or sunitinib (10 µM) for 2 hr. Following 2 hr of incubation, cells were washed with fresh medium, and incubated with drug-free medium for an additional 2 hr. During these 4 hr of incubation TFEB-eGFP was visualized by an InCell Analyzer fluorescence microscope. **b** The ratio between nuclear and cytoplasmic TFEB-eGFP levels was determined by staining nuclei with Hoechst 33342 (2 µg/ml) prior to drug exposure (nuclear staining is not shown), and image analysis by InCell investigator software. *p value < 0.05, **p value < 0.01—two-tailed Student's *t* test. Error bars denote standard deviation

To determine whether this drug-induced nuclear TFEB translocation is reversible, U2OS-TFEB-eGFP cells were treated with siramesine, sunitinib and Torin-1 for 2 hr, to induce nuclear TFEB translocation. Following 2 hr of drug exposure, cells were washed, and incubated in drug-free medium for an additional period of 2 hr. During this 4 hr incubation, TFEB-eGFP localization was visualized using time-lapse fluorescence microscopy (Fig. 6). Removal of the mTOR inhibitor Torin-1 from the growth medium resulted in a rapid export of TFEB from the nucleus to the cytoplasm; as early as 30 min after Torin-1 removal from the medium, a dominant cytoplasmic TFEB localization was restored. Siramesine-induced nuclear TFEB translocation was also reversible, however, the export time of TFEB into the cytoplasm after siramesine removal from the growth medium was slower, and even after 2 hr of drug removal the nuclear TFEB levels were significantly higher compared to untreated cells. Intriguingly, not only that sunitinib removal from the growth medium did not result in the export of TFEB to the cytoplasm, but TFEB continued accumulating in the nucleus even after 2 hr of drug removal.

**Fig. 7:**
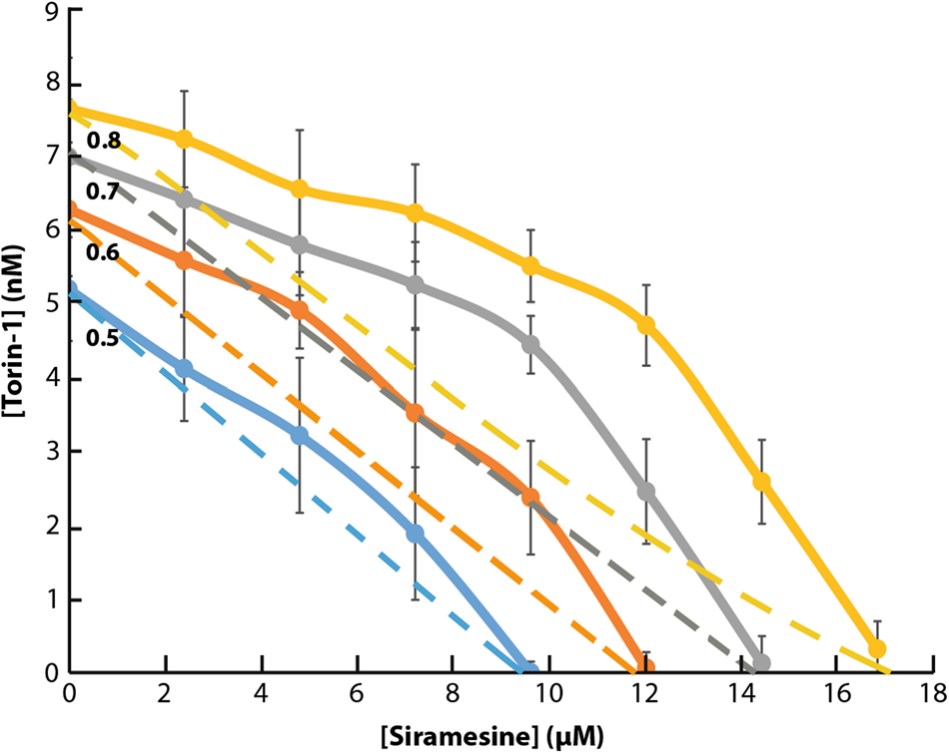
Isobologram analysis of the combined impact of Torin-1 and siramesine on nuclear translocation of TFEB. U2OS-TFEB-eGFP cells were treated for 3 hr with increasing concentrations of Torin-1 (1.2–8.4 nM) or siramesine (2.4–16.8 µM) and the combinations of the two. TFEB-eGFP was visualized using an InCell Analyzer fluorescence microscope. The ratio between nuclear and cytoplasmic TFEB-eGFP levels was determined by staining nuclei with Hoechst 33342 (2 µg/ml) prior to drug exposure, and image analysis by InCell investigator software. Dashed lines indicate the line of additivity for each effect (relative TFEB nuclear levels of 0.5, 0.6, 0.7, and 0.8). Full lines indicate experimental results of the drug combinations for the same effect level. Error bars indicate standard deviation

An isobologram analysis was performed to explore drug interaction between siramesine and Torin-1 by evaluating  inhibition of mTORC1, in regards to TFEB activation. To this end, U2OS-TFEB-eGFP cells were exposed to increasing concentrations of siramesine alone, Torin-1 alone, or combinations of the two compounds. Cells were incubated for 3 hr with these drugs, followed by imaging of TFEB-eGFP localization using fluorescence microscopy and computational image quantification. This isobologram analysis demonstrated a slightly sub-additive affect between the two compounds, as evident from the location of the isoboles above the lines of additivity across the different effect levels (Fig. 7). This result suggests that both siramesine and Torin-1 induce nuclear TFEB translocation via a similar mechanism, both inhibiting mTORC1 activity. We postulate that if siramesine was to activate TFEB via a pathway unrelated to mTORC1 inhibition, such as activation of TFEB dephosphorylation, a synergistic effect would be apparent in this analysis.

## Discussion

Our current findings constitute the first demonstration of lysosomal membrane fluidization by lysosomotropic anticancer drugs. Unlike the bona fide membrane fluidizer dibucaine, the LDs siramesine and sunitinib induced membrane fluidization only when the lumen of the liposomes was acidic, mimicking acidic lysosomes. From a mechanistic perspective, we have previously suggested that due to their hydrophobic weakly basic nature, these compounds reside within the hydrocarbon core of the lipid bilayer via their multi-aromatic ring structure, whereas the basic amine residue(s) of the molecule is presumably entrapped in a positively charged state within the acidic lumen of lysosomes^7^. Given the excessive accumulation of LDs in lysosomes^2,4,5,26^, they may exert membrane fluidization, hence impairing the function of central lysosomal membrane residents or protein complexes associated to the lysosomal membrane like mTORC1. Our findings are in accord with previous results with siramesine which was found to exert LMP, lysosomal leakage, and generation of reactive oxygen species^18,19^. Furthermore, previous studies have shown that siramesine binds with high affinity the acidic phospholipid phosphatidic acid in the lipid bilayer surface with a remarkable stoichiometry of 1:1^27^. Hence, these LDs will exert their membrane fluidization activity solely on biomembranes of acidic organelles like lysosomes, while having no apparent impact on other cellular membranes.

We herein demonstrated that LDs inhibit the activity of mTORC1 via membrane fluidization. We further show that mTORC1 is released from the lysosomal membrane into the cytosol following treatment with the LDs siramesine and sunitinib. It has been established that the activation of mTORC1 is dependent on its lysosomal localization and that the release of mTORC1 from the lysosomal membrane results in markedly reduced mTORC1 activity^22,28,29^. We thus postulate that release of mTORC1 from the lysosomal membrane following drug-induced membrane fluidization is the driving force behind mTORC1 inhibition. mTORC1 is recruited to the lysosomal surface by Rag guanosine triphosphatases (GTPases), and is activated therein via its interaction with the small GTPase Rheb^30,31^. It is therefore possible that lysosomal membrane fluidization reduces the activation of mTORC1 via interference with the ability of Rag GTPases to recruit mTORC1 to the lysosomal surface, inhibition of Rheb-mediated mTORC1 activation or possibly via inhibition of additional lysosomal co-factors which interact with mTORC1. In this context, it is well-established that the biophysical properties of a biomembrane not only regulate the activity of proteins embedded in it, but also the recruitment and activity of peripheral, amphitropic membrane proteins that bind weakly and reversibly to membrane lipids and are only temporarily associated to the membrane^32^. For example, we have previously shown that multidrug resistance chemosensitizers markedly enhance membrane fluidity resulting in inhibition of the ATPase activity of the multidrug efflux pump P-glycoprotein^33,34^. mTORC1 plays a crucial role in driving cell growth by regulating various processes of protein synthesis and degradation. mTORC1 functions as a downstream effector for multiple frequently mutated oncogenic pathways including the PI3K/Akt pathway and the Ras/Raf/Mek/Erk (MAPK) pathway, resulting in mTORC1 hyperactivation in a high percentage of human cancers^14,30,35,36^. Thus, LD-induced inhibition of mTORC1 might have therapeutic implications by inhibiting cancer cell growth, apart from the activation of TFEB and the consequent increase in lysosomal biogenesis, lysosomal exocytosis, and autophagy.

Herein, we showed that inhibition of mTORC1 is necessary for the activation and nuclear TFEB translocation. Since mTOR was found to be hyperactivated in a spectrum of human malignancies, numerous mTOR inhibitors are being developed as potential anticancer drugs^36^. While using mTOR inhibitors as potential anticancer drugs, this effect of mTORC1 inhibition on TFEB activation has to be taken into account, as the consequent activation of lysosomal biogenesis and lysosomal exocytosis might limit the efficiency of other chemotherapeutic agents with lysosomotropic properties due to their lysosomal sequestration and extrusion from the cell^5,6^.

The instantaneous response of TFEB to mTORC1 inhibition, evident as early as 12 min after cell exposure to Torin-1, suggests that TFEB is constitutively dephosphorylated and is retained in the cytoplasm due to constitutive rephosphorylation by mTORC1. Upon inhibition of mTORC1, this equilibrium is impaired, and the rapid dephosphorylation of TFEB results in its translocation into the nucleus. These results further demonstrate that LD-induced dephosphorylation and activation of TFEB is independent of cytosolic Ca^2+^ levels^9^. It is noteworthy that the reports of calcineurin acting as the phosphatase responsible for TFEB activation also presented only partial inhibition of TFEB activation when calcineurin activity was blocked^9^. Our results suggest that an additional phosphatase(s) may contribute to the activation of TFEB. Furthermore, our results demonstrate that after relieving mTORC1 inhibition, TFEB rapidly regains its cytosolic localization. Since TFEB phosphorylation by mTORC1 occurs on the lysosomal membrane, this result indicates that dephosphorylated TFEB constantly shifts between the nucleus and the cytoplasm, and can thus rapidly respond to changes in the state of mTORC1 activity.

**Fig. 8:**
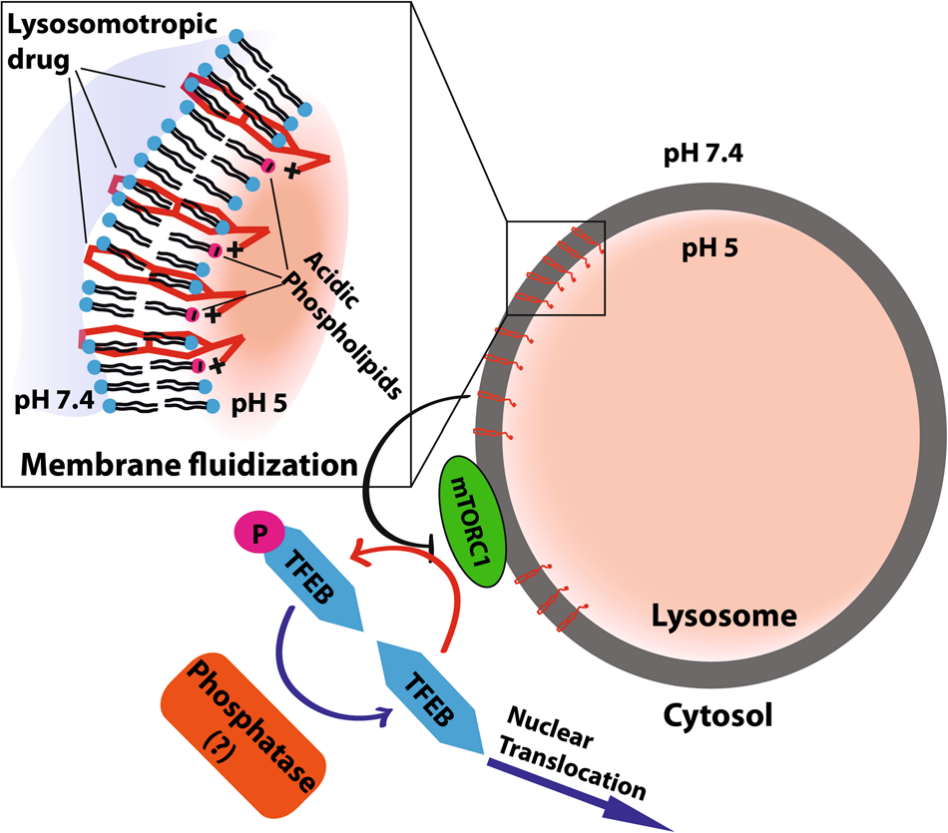
A proposed integrative model for drug-induced lysosomal membrane fluidization and consequent activation of TFEB due to mTORC1 inhibition. LDs diffuse freely into the cell, when reaching the lysosomal membrane these drugs intercalate into the lysosome membrane via their hydrophobic polyromantic ring structure with their amine group(s) presumably exposed to the acidic lumen of the lysosomes. These lysosomotropic compounds undergo efficient protonation, becoming positively charged and thus undergo high affinity interaction with acidic phospholipids in the lysosomal membrane including phosphatidic acid, thereby reaching a remarkable stoichiometry of 1:1^27^. This accumulation of amphiphilic compounds induces lysosomal membrane fluidization and consequent disruption of mTORC1 kinase activity. This in turn leads to loss of TFEB phosphorylation. TFEB dephosphorylation by a yet unknown phosphatase(s) that is distinct of calcineurin results in translocation of dephosphorylated TFEB to the nucleus. This culminates in CLEAR gene network induction and lysosomal biogenesis

Based on the current cumulative findings and the data from previous papers, we herein propose an integrative model for drug-induced lysosomal membrane fluidization, which leads to inhibition of mTORC1 kinase activity, resulting in a shift in the equilibrium of TFEB toward a dephosphorylated state, thereby inducing TFEB translocation to the nucleus and consequent activation of the CLEAR gene network (Fig. 8).

## Materials and methods

### Chemicals

Siramesine, chloroquine, cyclosporin A, FK-506 (tacrolimus) and Hoechst 33342 were obtained from Sigma-Aldrich (St. Louis, MO, USA). BAPTA-AM was obtained from Biotium, Inc. (Fremont, CA, USA). Torin-1 was purchased from BioVision, Inc. (Milpitas, CA, USA). Sunitinib was a kind gift from Prof. A.W. Griffioen, VU Medical Center, Amsterdam, The Netherlands. 1,2-dioleoyl-sn-glycero-3-phosphocholineall (DOPC), 1,2-distearoyl-sn-glycero-3-phosphoethanolamine-N-[biotinyl(polyethylene glycol)-2000] (DSPE-PEG(2000) Biotin), 1,2-dioleoyl-sn-glycero-3-phosphoethanolamine-N-(lissamine rhodamine B sulfonyl) (18:1 Liss-Rhod-PE) and cholesterol were obtained from Avanti Polar Lipids, (Alabaster, AL, USA). 1,2-Distearoyl-sn-glycero-3-phosphoethanolamine (DSPE) were purchased from Echelon Biosciences Inc, (Salt Lake City, UT, USA). Streptavidin-coated 2 µm polystyrene microspheres were purchased from Spherotech (Lake forest, Illinois, USA).

### Liposome preparation and FRAP analysis

Uniform microsphere-supported bilayer membranes were created via adsorption of liposomes to polystyrene microspheres^37^. The use of streptavidin-coated microspheres and biotin-labeled lipids with a PEG-2000 linker, results in a “hydration layer” that preserves the fluidity of the membrane^38^, and allows creating an interior environment that differs from the surrounding one. In order to mimic the acidic lysosome lumen, we entrapped inside the supported membrane an acidic solution (pH 5), while in the extravesicular milieu we maintained a neutral pH solution (pH 7.4) reflecting the cytoplasmic pH.

Single unilamellar vesicles (SUVs) were prepared in a glove box where the atmosphere containing O_2_ was exchanged three times with N_2_. Totally, 300 nmol of lipids with a composition that resembles that of lysosomes^20^ (molar ratio of DOPC:DSPE:Cholesterol:DSPE-PEG(2000)-Biotin:Liss-Rhod-PE 0.375:0.244:0.3505:0.02:0.005) were mixed in a clean glass vial from chloroform stock solutions. The chloroform lipid mixture was dried under a gentle stream of N_2_ for 30 min while the glass vial was slowly rotated, and further dried under vacuum (SpeedVac, Labconco, USA) for 2 hr. The dry lipid film was hydrated with 10 mM Tris-HCl, 150 mM NaCl at pH 7.4 or, to mimic the lumen of the lysosome, at pH 5.0. Hydrated lipid films were then left at 4 °C overnight, vortexed (speed 8 in ZX3, VELP Scientifica, Italy) for 1 hr and sonicated in a bath sonicator (S10H elmasonic, ELMA, Germany) for an additional 1 hr. A homogeneous population of small unilamellar vesicles was formed by repeated (40 times) extrusion through 100 nm polycarbonate filters using an extruder (Avanti Polar Lipids, USA).

Coating of the microspheres was achieved by mixing equal volumes (50 µL) of SUVs (0.6 mM) and freshly cleaned 2 µm microspheres 0.6% (W/V) with 100 µL of 10 mM Tris-HCl, 150 mM NaCl at pH 7.4 or pH 5.0 in a 1.5 ml Eppendorf test tube. The tube was placed in a bath sonicator for 15 min sonication on ice, and then gently mixed on a rocker (program F8 speed 50 in Intelli-Mixer RM-2L, Elmi, Russia) for 2 hr. Excess vesicles were removed by washing three times with the same buffer using a centrifuge (5430R centrifuge, Eppendorf, Germany). At the last washing step, the microspheres were precipitated with 10 mM Tris-HCl, 150 mM NaCl, at pH 7.4.

The integrity of the microsphere’s membrane coverage was verified using a confocal microscope (Confocal Zeiss LSM 700) with a 63×, NA 1.4, PlanApo oil immersion objective on an IX81 inverted microscope. Incorporation of 0.5% molar of Rhodamine head group labeled lipids (18:1 Liss-Rhod-PE) in the preparation of the SUVs enables imaging of the membrane on the microspheres using excitation at 555 nm. Fluorescence images were taken along with the corresponding bright field, diffusion interference contrast image.

For FRAP measurements, the integrated settings in the Zeiss confocal software were utilized. In a typical procedure, a random microsphere was chosen and the image was centered on it and digitally zoomed in by ×10. Bleaching of a section of the liposome was performed using a single iteration of a high intensity laser pulse (100%). The fluorescence intensity at the photobleached area was then recorded over time, as the fluorescent lipids diffuse on the surface of the sphere (Fig. 1a). For FRAP analysis, a reference non-bleached area of the microsphere was chosen and compared to the bleached area. The fluorescence recovery time, *T*_f_, was determined by fitting an exponential function to the fluorescence intensity, *I*, at the photobleached region over time:

$${I}\left( {t} \right) = {I}_\infty \left( {1 - {e}^{ - \frac{{t}}{{{T}_{\mathrm{f}}}}}} \right){.}$$

### Cell culture and stable transfections

U2OS human osteosarcoma, MCF-7 breast cancer and HeLa cervical cancer cells used in this paper were maintained in RPMI-1640 medium (Gibco, Paisley, UK), supplemented with 10% fetal bovine serum, 2 mM glutamine, 100 μg/ml penicillin and streptomycin (Biological Industries, Beit HaEmek, Israel) in a humid atmosphere containing 5% CO_2_ at 37 °C. U2OS and MCF-7 cells were transfected with TFEB-eGFP using Linear Polyethylenimine (PEI, MW 25,000) transfection reagent (Polysciences, Pennsylvania, USA) at a ratio of 3 µg PEI: 1 µg DNA. For stable transfections, 34 hr after transfection, cells were subjected to G-418 selection (700 μg/ml; Sigma-Aldrich, St. Louis, MO, USA) in the growth medium. pEGFP-N1-TFEB was a gift from Shawn Ferguson (Addgene plasmid # 38119)^13^.

### Protein extraction and WB analysis

For WB analysis of the phosphorylation levels of the various proteins, cells were plated on 100 mm plates, and allowed to attach for 48 hr. Cells were then treated with the above described drugs for 3 hr. Illumination of sunitinib-treated cells, where stated, was performed as previously described^4^. Cytosolic and nuclear proteins were extracted as previously described^39^ and protein content was determined using the Bio-Rad protein assay (Bio-Rad, Hercules, CA, USA). All WB were performed using 10% acrylamide gels. For evaluating the levels of p70 S6K phosphorylation cytosolic (50 µg) and nuclear (30 µg) proteins were used, while cytosolic (30 µg) and nuclear (15 µg) proteins were used for the detection of Elk-1 and its phosphorylated form. The antibodies for Elk-1, phospho-Elk-1, p70-S6K, and phospho-p70-S6K were purchased from Cell Signaling (Danvers, MA, USA), and were used at a dilution of 1:1000. A β-actin-specific antibody was used to assess actual protein loading (Sigma-Aldrich St. Louis, MO, USA; dilution 1: 10,000). The membrane was then reacted with horseradish-peroxidase conjugated secondary antibodies (Jackson Immunoresearch Labs, West Grove, PA) and enhanced chemiluminescence (ECL) detection was performed according to the manufacturer’s instructions (Biological Industries, Beit HaEmek, Israel). Membranes were exposed for increasing times and the images chosen for band quantification by ImageJ software were carefully verified for linear range band intensity. Phosphorylation levels were determined by dividing the level of phosphorylated protein by the levels of total protein for each treatment.

For the detection of TFEB, U2OS cells were transiently transfected with a TFEB-3xFLAG construct (kindly provided by Prof. A. Ballabio, Telethon Institute of Genetics and Medicine, Naples, Italy) as described above. 24 hr after transfection, cells were exposed to the indicated drugs for 3 hr followed by protein extraction. Cytosolic (30 µg) and nuclear (15 µg) proteins were loaded and an anti-FLAG antibody (Sigma Aldrich, St. Louis, MO, USA) was used. To ensure a good separation between cytosolic and nuclear proteins and to confirm equal protein loading, the membrane was stripped and reacted with anti-calreticulin (Sigma Aldrich) or anti-SP1 (Santa Cruz, Dallas, TX, USA) antibodies.

### Immunofluorescence assays

Immunofluorescence assays were performed as follows: U2OS cells were seeded in 24-well plates on sterile glass coverslips. After 48 hr, cells were incubated with either vehicle (0.1% DMSO), 10 nM Torin1, 10 µM siramesine or 10 µM sunitinib for 3 hr. Cells treated with sunitinib, were either kept in the dark or exposed to light for 1 hr, as previously described^4^. Cells were then washed twice with PBS, fixed with a fresh solution of 4% formaldehyde in PBS for 15 min and washed twice for 5 min in PBS. Permeabilization was then performed using 0.1% Triton X-100 in PBS for 10 min followed by two washes with PBS. Cells were incubated for 1 hr at room temperature (RT) in TBS buffer (10 mM Tris, 150 mM NaCl, pH 7.4) containing 20% skim milk, and then incubated for 1 hr at RT with primary antibodies against LAMP1 (sc-20011, Santa Cruz Biotechnology, Dallas, TX, USA) and mTOR (#2972, Cell Signaling Technology, Danvers, MA, USA). Following three 5 min washes with PBS, cells were co-incubated with Rhodamine Red Donkey anti-mouse and Alexa Fluor-488 Donkey anti-rabbit secondary antibodies (Jackson Immunoresearch, West Grove, PA, USA) along with 1 µg/ml Hoechst 33342 (Sigma Aldrich, St. Louis, MO, USA) for 1 hr at RT in the dark. Cells were then washed three times in PBS for 5 min and the coverslips were mounted onto microscope slides over fluoromount-G (Thermo Fisher Scientific, Waltham, MA, USA). Fluorescence was recorded using a confocal *Zeiss* LSM 710 microscope (×63 magnification), using the same laser intensities and detector gain for all slides in the experiment. Image processing was performed using the *Zeiss* Black edition software.

### Live cell imaging

Stably transfected U2OS-TFEB-eGFP and MCF-7-TFEB-eGFP cells were plated at 40% confluence in 24-well glass bottom plates (In Vitro Scientific, CA, USA). For TFEB-eGFP subcellular localization studies, cells were exposed to siramesine (10 µM), sunitinib (10 µM), chloroquine (100 µM), or mefloquine (10 µM) for the duration of the experiment. TFEB-eGFP fluorescence was followed with a fluorescence microscope InCell analyzer 2000 (GE Healthcare Bio-Sciences, Pittsburgh, PA, USA). Computational analysis of TFEB translocation into the nucleus was determined using InCell investigator software. To achieve nuclear staining prior to fluorescence imaging, cells were incubated with 2 μg/ml Hoechst 33342 in growth medium for 10 min. In TFEB-eGFP localization experiments where BAPTA-AM was used, cells were preincubated with BAPTA-AM (10 µM) for 30 min and washed with fresh medium prior to drug exposure. In TFEB-eGFP localization experiments with calcineurin inhibitors, cells were co-incubated with the stated drugs with or without CSA (10 µM), FK-506 (5 µM), or both for the duration of the experiment.

For Ca^2+^ release experiments, U2OS cells were plated in 24-well glass bottom plates, preincubated with the Ca^2+-^dependent stain Fluo-8-AM (3 µg/ml) (AAT Bioquest, Inc. Sunnyvale, CA, USA) for 30 min, washed with fresh medium, and incubated with or without siramesine (10 µM) or chloroquine (100 µM) for 15 min.

For isobologram experiments, U2OS-TFEB-eGFP cells were plated at 40% confluence in 96-well plates, and incubated overnight to allow attachment. Cells were incubated with increasing concentrations of siramesine (2.4–16.8 μM) or Torin-1 (1.2–8.4 nM) or combinations of the two compounds, and incubated for 3 hr. Following incubation, nuclei were stained by adding Hoechst 33342 (2 μg/ml) to the growth medium. TFEB-eGFP and nuclei were visualized by an InCell Analyzer 2000 fluorescence microscope. Computational analysis of TFEB translocation into the nucleus was determined using InCell investigator software.

### RNA extraction and quantification of lysosomal gene expression by real-time PCR

U2OS cells were seeded in 6-well plates and incubated for 48 hr at 37 °C. Cells were then treated with siramesine (10 μM), sunitinib (10 μM) or chloroquine (100 μM) for 4, 8, or 16 hr. RNA extraction and cDNA synthesis were carried out as previously described^40^. Gene expression levels of V-type proton ATPase subunit HATPV1H (ATP6V1H), glucosamine (N-Acetyl)-6-Sulfatase (GNS), and cathepsin D (CTSD) were determined using a quantitative real-time PCR assay as previously described^40^. Gene expression levels were normalized using the β-glucuronidase (GUSB) gene as an internal control. The primers used for real-time PCR were: ATP6V1H—AGCCCTGAAGAGAAGCAAGAGA, CGATTCAACATTGGCAGAAAGT; GNS—CCCATTTTGAGAGGTGCCAGT, TGACGTTACGGCCTTCTCCTT; CTSD—TGCTCAAGAACTACATGGACGC, CGAAGACGACTGTGAAGCACT; GUSB—CCATTCCTATGCCATCGTG, ATGTCGGCCTCGAAGGG.

### Statistical analysis

Three or more biological repeats were performed for each experiment described. Two-tailed student’s *t* test was used to determine the significance of the results when comparing band intensity in WB and recovery time in FRAP experiments. In live cell imaging experiments the quantification of nuclear translocation of TFEB was performed by sampling fields with at least 20 cells per field. The significance of the difference in nuclear TFEB levels was determined at each time point by a two-tailed student’s *t* test.

## Electronic supplementary material

Supplementary Figure S1

Supplementary Figure S2

Supplementary Figure S3

Supplementary Video 1a

Supplementary Video 1b

Supplementary Video 1c

Supplementary Video 1d

Supplementary Video 1e

supplementary figure legends

## References

[1] Kaufmann AM, Krise JP (2007). Lysosomal sequestration of amine-containing drugs: analysis and therapeutic implications. J. Pharm. Sci..

[2] Zhitomirsky B, Assaraf YG (2016). Lysosomes as mediators of drug resistance in cancer. Drug Resist. Updat..

[3] Kazmi F (2013). Lysosomal sequestration (trapping) of lipophilic amine (cationic amphiphilic) drugs in immortalized human hepatocytes (Fa2N-4 cells). Drug Metab. Dispos..

[4] Adar Y (2012). Imidazoacridinone-dependent lysosomal photodestruction: a pharmacological Trojan horse approach to eradicate multidrug-resistant cancers. Cell Death Dis..

[5] Zhitomirsky B, Assaraf YG (2015). Lysosomal sequestration of hydrophobic weak base chemotherapeutics triggers lysosomal biogenesis and lysosome-dependent cancer multidrug resistance. Oncotarget.

[6] Zhitomirsky, B. & Assaraf, Y. G. Lysosomal accumulation of anticancer drugs triggers lysosomal exocytosis. *Oncotarget*. 10.18632/oncotarget.15155 (2017) 10.18632/oncotarget.15155 PMC5542171 28187461

[7] Zhitomirsky B, Assaraf YG (2015). The role of cytoplasmic-to-lysosomal pH gradient in hydrophobic weak base drug sequestration in lysosomes. Cancer Cell Microenviron..

[8] Sardiello M (2009). A gene network regulating lysosomal biogenesis and function. Science..

[9] Medina DL (2015). Lysosomal calcium signalling regulates autophagy through calcineurin and TFEB. Nat. Cell Biol..

[10] Settembre C, Fraldi A, Medina DL, Ballabio A (2013). Signals from the lysosome: a control centre for cellular clearance and energy metabolism. Nat. Rev. Mol. Cell Biol..

[11] Medina DL (2011). Transcriptional activation of lysosomal exocytosis promotes cellular clearance. Dev. Cell..

[12] Palmieri M (2011). Characterization of the CLEAR network reveals an integrated control of cellular clearance pathways. Hum. Mol. Genet..

[13] Roczniak-Ferguson, A. et al. The transcription factor TFEB links mTORC1 signaling to transcriptional control of lysosome homeostasis. *Sci. Signal*. 10.1126/scisignal.2002790 (2012) 10.1126/scisignal.2002790 PMC3437338 22692423

[14] Laplate M, Sabatini DM (2012). mTOR signaling in growth control and disease. Cell.

[15] Settembre C (2012). A lysosome-to-nucleus signalling mechanism senses and regulates the lysosome via mTOR and TFEB. EMBO J..

[16] Martina JA, Puertollano R (2013). Rag GTPases mediate amino acid-dependent recruitment of TFEB and MITF to lysosomes. J. Cell Biol..

[17] Martina JA, Chen Y, Gucek M, Puertollano R (2012). MTORC1 functions as a transcriptional regulator of autophagy by preventing nuclear transport of TFEB. Autophagy.

[18] Ostenfeld MS (2005). Effective tumor cell death by σ-2 receptor ligand siramesine involves lysosomal leakage and oxidative stress. Cancer Res..

[19] Ostenfeld MS (2008). Anti-cancer agent siramesine is a lysosomotropic detergent that induces cytoprotective autophagosome accumulation. Autophagy.

[20] Olsson JM, Daliner G (1991). Lipid compositions of intracellular membranes isolated from rat liver nodules in Wistar rats. Cancer Res..

[21] Nowak-Sliwinska P (2015). Photoactivation of lysosomally sequestered sunitinib after angiostatic treatment causes vascular occlusion and enhances tumor growth inhibition. Cell Death Dis..

[22] Sancak Y (2008). The rag GTPases bind raptor and mediate amino acid signaling to mTORC1. Science.

[23] Sancak Y (2010). Ragulator-rag complex targets mTORC1 to the lysosomal surface and is necessary for its activation by amino acids. Cell.

[24] Thoreen CC (2009). An ATP-competitive mammalian target of rapamycin inhibitor reveals rapamycin-resistant functions of mTORC1. J. Biol. Chem..

[25] Sugimoto T, Stewart S, Guan KL (1997). The calcium/calmodulin-dependent protein phosphatase calcineurin is the major Elk-1 phosphatase. J. Biol. Chem..

[26] Gotink KJ (2011). Lysosomal sequestration of sunitinib: a novel mechanism of drug resistance. Clin. Cancer Res..

[27] Parry MJ (2008). High-affinity small molecule-phospholipid complex formation: binding of siramesine to phosphatidic acid. J. Am. Chem. Soc..

[28] Li Z (2016). Ammonia induces autophagy through Dopamine receptor D3 and MTOR. PLoS One.

[29] Betz C, Hall MN (2013). Where is mTOR and what is it doing there?. J. Cell Biol..

[30] Zoncu R, Sabatini DM, Efeyan A (2012). mTOR: from growth signal to diabetes and cancer. Nat. Rev. Mol. Cell Biol..

[31] Zoncu R (2011). mTORC1 senses lysosomal amino acids through an inside-out mechanism that requires the vacuolar H+–ATPase. Science.

[32] Mouritsen OG (2011). Lipidology and lipidomics––quo vadis? A new era for the physical chemistry of lipids. Phys. Chem. Chem. Phys..

[33] Drori S, Eytan GD, Assaraf YG (1995). Potentiation of anticancer‐drug cytotoxicity by multidrug‐resistance chemosensitizers involves alterationsin membrane fluidity leading to increased membrane permeability. Eur. J. Biochem..

[34] Regev R, Assaraf YG, Eytan GD (1999). Membrane fluidization by ether, other anesthetics, and certain agents abolishes P-glycoprotein ATPase activity and modulates efflux from multidrug- resistant cells. Eur. J. Biochem..

[35] Guertin DA, Sabatini DM (2007). Defining the role of mTOR in cancer. Cancer Cell..

[36] Xie J, Wang X, Proud CG (2016). mTOR inhibitors in cancer therapy. F1000Research.

[37] Gopalakrishnan G, Rouiller I, Colman DR, Lennox RB (2009). Supported bilayers formed from different phospholipids on spherical silica substrates. Langmuir.

[38] Rädler J, Strey H, Sackmann E (1995). Phenomenology and kinetics of lipid bilayer spreading on hydrophilic surfaces. Langmuir.

[39] Schreiber E, Matthias P, Müller MM, Schaffner W (1989). Rapid detection of octamer binding proteins with “mini extracts”, prepared from a small number of cells. Nucleic Acids Res..

[40] Raz S (2014). Severe hypoxia induces complete antifolate resistance in carcinoma cells due to cell cycle arrest. Cell Death Dis..

