# Supplementary figures and images for: Lysosomotropic drugs activate TFEB via lysosomal membrane fluidization and consequent inhibition of mTORC1 activity
Source: Cell Death Dis. 2018 Dec 13;9(12):1191. doi: 10.1038/s41419-018-1227-0 (PMC6294013; doi:10.1038/s41419-018-1227-0)

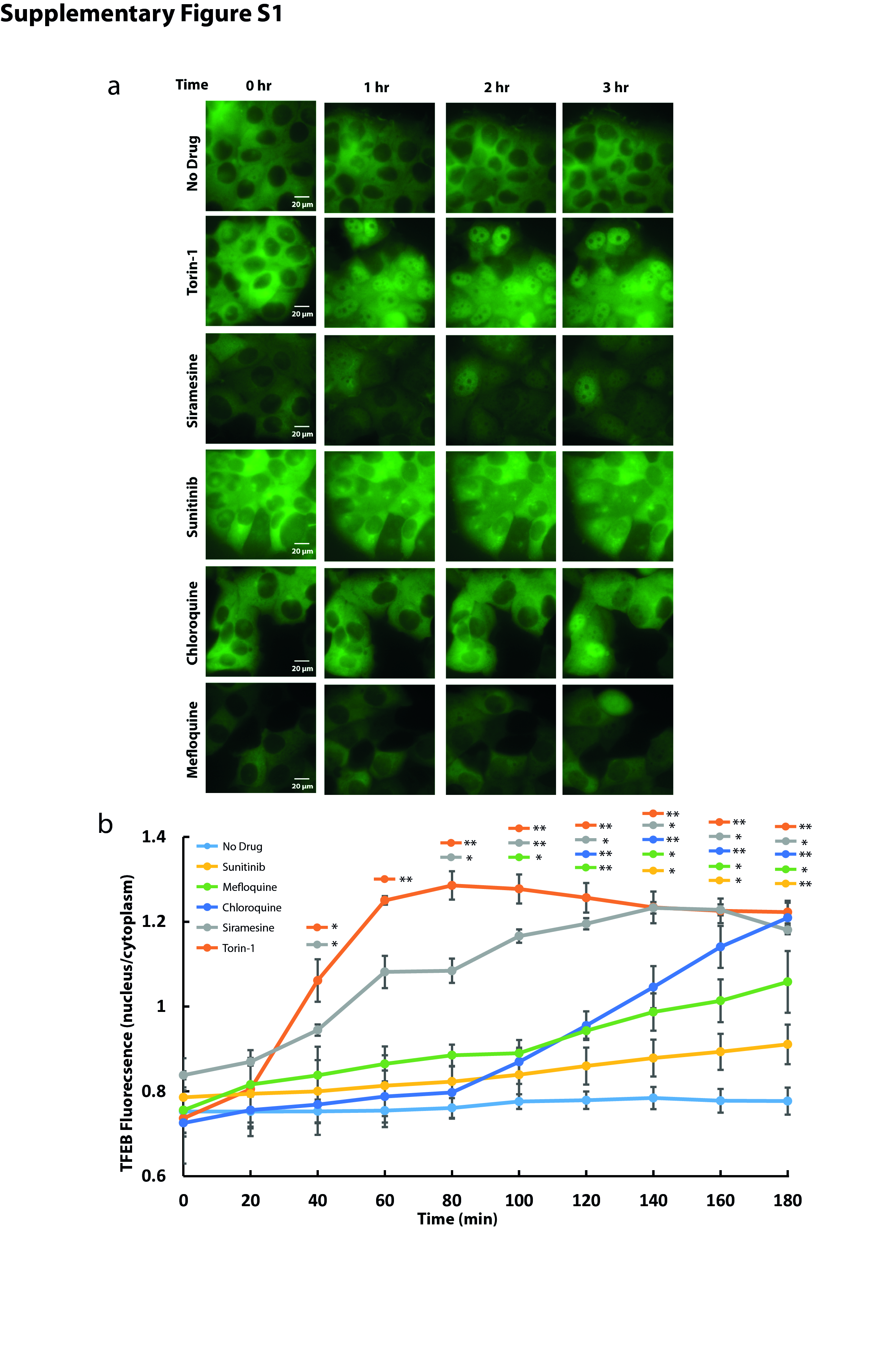

Supplement: Supplementary file 1 — Supplementary Figure S1 [file 41419_2018_1227_MOESM1_ESM.tif]

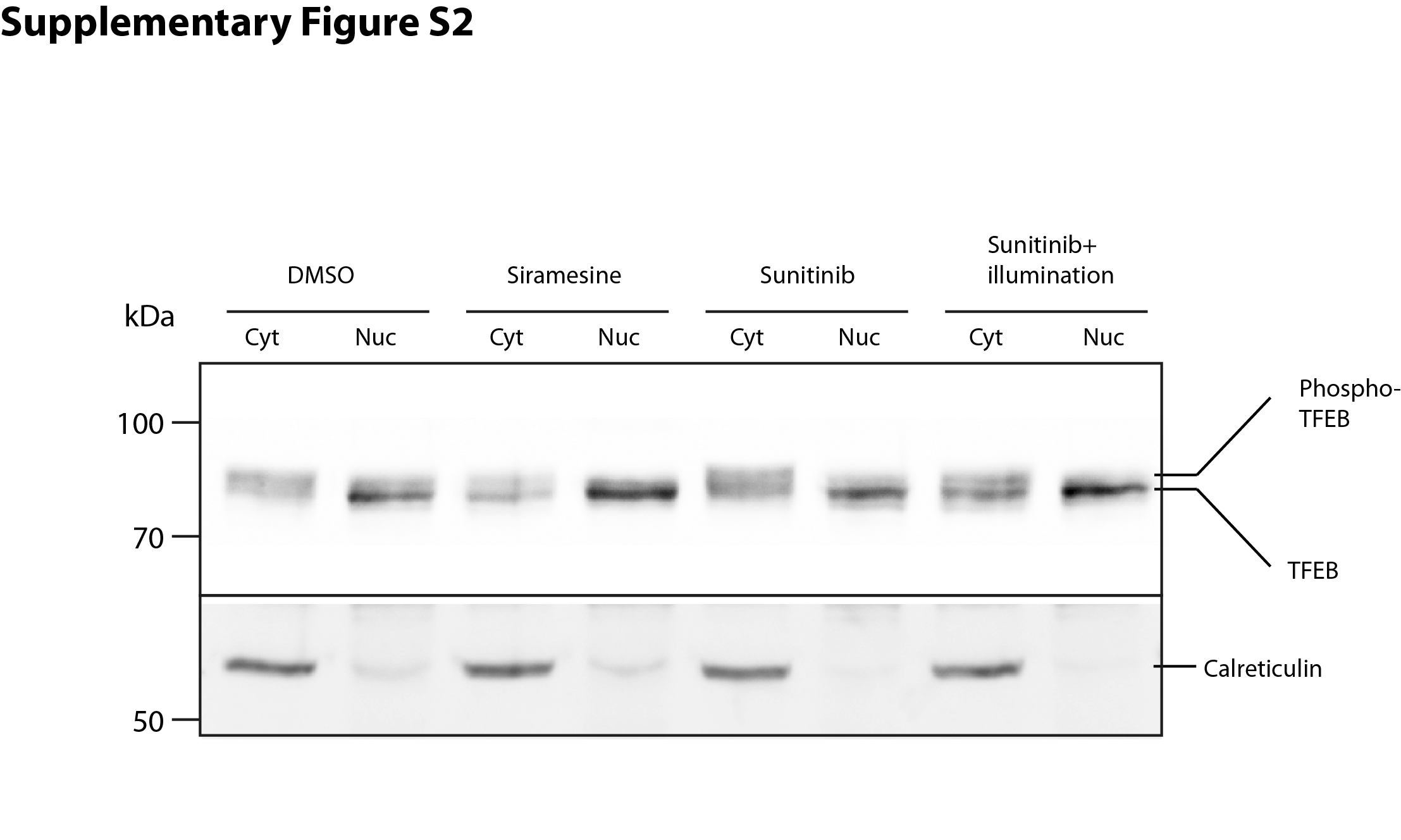

Supplement: Supplementary file 2 — Supplementary Figure S2 [file 41419_2018_1227_MOESM2_ESM.tif]

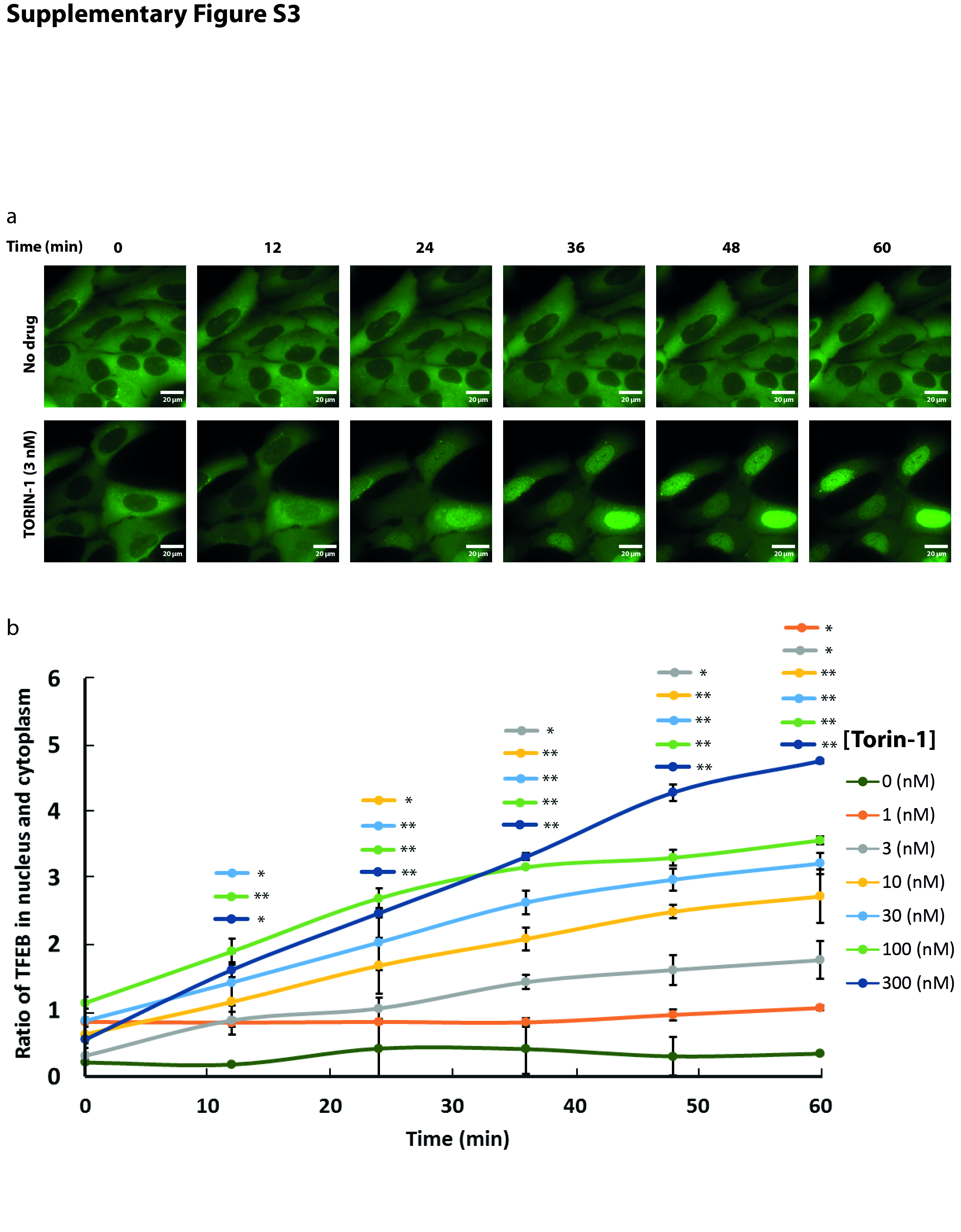

Supplement: Supplementary file 3 — Supplementary Figure S3 [file 41419_2018_1227_MOESM3_ESM.tif]
