# Supplementary material for: Lysosomotropic drugs activate TFEB via lysosomal membrane fluidization and consequent inhibition of mTORC1 activity
Source: Cell Death Dis. 2018 Dec 13;9(12):1191. doi: 10.1038/s41419-018-1227-0 (PMC6294013; doi:10.1038/s41419-018-1227-0)
Supplement: Supplementary file 9 — supplementary figure legends [file 41419_2018_1227_MOESM9_ESM.docx]

**Supplementary Fig. S1**: **Lysosomotropic drugs induce a rapid translocation of TFEB into the nucleus and activation of the CLEAR network.** MCF-7-TFEB-eGFP cells were incubated with Torin-1 (10 nM), siramesine (10 µM), sunitinib (10 µM), chloroquine (100 µM) or mefloquine (10 µM) for 3 hr. TFEB-eGFP was visualized by an InCell Analyzer fluorescence microscope every 20 min (a). The ratio between nuclear and cytoplasmic TFEB-eGFP levels was determined by staining nuclei with Hoechst 33342 prior to drug exposure (2 µg/ml, staining data not shown), and image analysis by InCell investigator software (b). *p-value <0.05, ** p-value<0.01 – two tailed Students t-test. Error bars indicate standard deviation.

**Supplementary Fig. S2: Drug treatment induces a lower molecular weight TFEB which is translocated to the nucleus**. TFEB-x3FLAG transfected cells were treated with the indicated drugs for 3 hr and harvested for nuclear and cytosolic protein extraction as detailed in the Materials and Method section. TFEB was detected using an ani-FLAG antibody (a) and following stripping of the membrane, it was reacted with anti-calreticulin (b) or an anti-SP1 (c) antibodies for the assessment of cytosolic and nuclear protein levels, respectively.

**Supplementary Fig. S3: Inhibition of mTORC-1 by Torin-1 results in a rapid, dose-dependent translocation of TFEB into the nucleus.** U2OS-TFEB-eGFP cells were treated with increasing concentrations of the mTOR inhibitor Torin-1 for 1 hr. TFEB-eGFP was visualized by InCell Analyzer fluorescence microscope every 12 min (a). The ratio between nuclear and cytoplasmic TFEB-eGFP levels was determined by staining nuclei with Hoechst 33342 prior to drug exposure (2 µg/ml, staining not shown), and image analysis by InCell investigator software (b). *p-value <0.05, ** p-value<0.01 – two tailed Students t-test. Error bars indicate standard deviation.

**Supplementary Videos 1a-e:** **Lysosomotropic drugs induce an elevation in cytosol Ca^2+^ levels.** U2OS cells were pre-stained with Fluo-8-AM (3 µg/ml, for 30 min). Then, Fluo-8-AM fluorescence indicative of Ca^2+^ levels, was visualized by InCell Analyzer fluorescence microscopy every 30 sec for 15 min without drug treatment (a), with siramesine (10 µM) (b), or with chloroquine (100 µM) (c). To chelate free Ca^2+^, U2OS cells were pre-treated with BAPTA-AM (10 µM, for 30 min) and pre-stained with Fluo-8-AM (3 µg/ml, for 30 min). Fluo-8-AM fluorescence in BAPTA-treated stained cells, indicative of Ca^2+^ levels, was visualized by an InCell Analyzer fluorescence microscope every 30 sec for 15 min with siramesine (10 µM) (d) or with chloroquine (100 µM) (e).
